# Effects of heat and drought stress on post‐illumination bursts of volatile organic compounds in isoprene‐emitting and non‐emitting poplar

**DOI:** 10.1111/pce.12643

**Published:** 2016-01-18

**Authors:** Werner Jud, Elisa Vanzo, Ziru Li, Andrea Ghirardo, Ina Zimmer, Thomas D. Sharkey, Armin Hansel, Jörg‐Peter Schnitzler

**Affiliations:** ^1^ Institute of Ion and Applied PhysicsUniversity of Innsbruck6020InnsbruckAustria; ^2^ Research Unit Environmental Simulation (EUS), Institute of Biochemical Plant Pathology (BIOP)Helmholtz Zentrum München GmbH85764NeuherbergGermany; ^3^ Department of Biochemistry and Molecular BiologyMichigan State UniversityEast Lansing Michigan48823USA

**Keywords:** *Populus* x *canescens*, acetaldehyde, climate change scenarios, green leaf volatiles, grey poplar, isoprene, LOX, post‐illumination bursts, VOC

## Abstract

Over the last decades, post‐illumination bursts (PIBs) of isoprene, acetaldehyde and green leaf volatiles (GLVs) following rapid light‐to‐dark transitions have been reported for a variety of different plant species. However, the mechanisms triggering their release still remain unclear. Here we measured PIBs of isoprene‐emitting (IE) and isoprene non‐emitting (NE) grey poplar plants grown under different climate scenarios (ambient control and three scenarios with elevated CO_2_ concentrations: elevated control, periodic heat and temperature stress, chronic heat and temperature stress, followed by recovery periods). PIBs of isoprene were unaffected by elevated CO_2_ and heat and drought stress in IE, while they were absent in NE plants. On the other hand, PIBs of acetaldehyde and also GLVs were strongly reduced in stress‐affected plants of all genotypes. After recovery from stress, distinct differences in PIB emissions in both genotypes confirmed different precursor pools for acetaldehyde and GLV emissions. Changes in PIBs of GLVs, almost absent in stressed plants and enhanced after recovery, could be mainly attributed to changes in lipoxygenase activity. Our results indicate that acetaldehyde PIBs, which recovered only partly, derive from a new mechanism in which acetaldehyde is produced from methylerythritol phosphate pathway intermediates, driven by deoxyxylulose phosphate synthase activity.

## Abstract 2

We investigated how combined heat and drought stress under elevated CO2 impact post illumination emissions of volatile organic compounds (VOCs) from isoprene emitting (IE) and isoprene non‐emitting (NE) poplar leaves. While in general stress is thought to induce the release of green leaf volatiles (GLV) we observed the opposite ‐ a suppression of GLV and acetaldehyde post illumination bursts (PIB) as well as a reduction in lipoxygenase (LOX) activity during heat and drought stress. The observed reduction of these short‐term stimulated pulses of GLV and acetaldehyde in environmentally challenged plants could be used as fast and non‐invasive markers in plant phenotyping applications.

## Introduction

Acetaldehyde and compounds commonly referred to as green leaf volatiles [GLVs, different C_6_ compounds originating from the lipoxygenase (LOX) pathway, such as E‐2‐hexenal, Z‐3‐hexenal and Z‐3‐hexenol], are among the most prominent oxygenated volatile organic compounds (oVOCs) in the earth's atmosphere (Guenther *et al.* 2012). Owing to indirect and direct sources from vegetation and microbial activity, they are frequently detected in different vegetative environments (e.g. Kesselmeier & Staudt 1999 and references therein).

Green leaf volatiles are often released in considerable amounts by plants under abiotic stress and herbivory or pathogen infection (Loreto & Schnitzler 2010; Holopainen & Gershenzon 2010; Scala *et al.* 2013). Their formation process in plant chloroplasts seems to be well understood nowadays: lipases are responsible for the release of linoleic acid (C18:2) and *α*‐linolenic acid (C18:3) from galactolipids of the thylakoid membranes. In a second step, lipoxygenase [9‐LOX and 13‐LOX (linoleate:oxygen 9‐ or 13‐oxidoreductase)] enzymes induce the degradation of these free fatty acids (Hatanaka 1993). Subsequent action of hydroperoxide lyase (HPL), alcohol dehydrogenase (ADH) and isomerization factors are eventually responsible for a distinct temporal succession of the GLVs released (Fall *et al.* 1999).

It could be shown that the same GLVs are also transiently released by different plant species after suddenly terminating illumination (Holzinger *et al.* 2000; Graus *et al.* 2004; Loreto *et al.* 2006; Brilli *et al.* 2011; Ghirardo *et al.* 2011; Jardine *et al.* 2012), indicating that a similar mechanism might be involved here. It has been argued that these so‐called ‘post‐illumination bursts’ (PIBs) might be caused by fast changes in intracellular pH values and consequently membrane stability after fast light‐to‐dark transitions (Brilli *et al.* 2011). In the algae *Eremosphaera viridis*, a quick light‐to‐dark transition leads to a short‐term (1–3 min) acidification of the cytosol (Bethmann *et al.* 1998). This effect could trigger the activation of LOX, which has its activity optimum at pH values of 6.3 and 4.5 (Hatanaka 1993). Conversely, fast dark‐to‐light transitions lead to an alkalization (Bethmann *et al.* 1998), which might explain why fast, repeated light‐to‐dark transitions do not cause PIBs, as has been shown previously using grey poplar leaves (Graus *et al.* 2004).

Experiments of Graus *et al.* (2004) and Jardine *et al.* (2012) revealed that GLV PIBs are strongly reduced when grey poplar leaves or mesquite (*Prosopis* spec.) branches, respectively, are kept under anoxic conditions. This is consistent with the supposed fatty acid degradation mechanism, because LOX activity requires oxygen.

Along with the GLVs, also acetaldehyde is emitted after leaf‐cutting, during drying or leaf collapse under high temperature (Fall *et al.* 1999; Holzinger *et al.* 2000; Brilli *et al.* 2011; Behnke *et al.* 2013); transient acetaldehyde emissions have been detected after root flooding (Kreuzwieser *et al.* 2000, 2001) and after light‐to‐dark transitions (Holzinger *et al.* 2000; Karl *et al.* 2002; Brilli *et al.* 2011; Ghirardo *et al.* 2011; Jardine *et al.* 2012). Acetaldehyde emissions after root flooding are explained by an interconversion of ethanol formed in anoxic roots or stems. Root ethanol is transported to the leaves by the transpiration stream and then oxidized to acetaldehyde (by ADH) and eventually by aldehyde dehydrogenase (ALDH) to acetate (Kreuzwieser *et al.* 1999, 2000).

Although there seems to be an apparent correlation between GLV and acetaldehyde emission after wounding and light‐to‐dark transitions, the underlying mechanisms are probably different. For acetaldehyde PIBs, some investigations were proposing a ‘pyruvate overflow mechanism’ (Karl *et al.* 2002; Hayward *et al.* 2004). Cytosolic pyruvate was thought to increase transiently, thus lowering the cytosolic pH, due to a decreased pyruvate import into the organelles after darkening. When a threshold pyruvate concentration is reached, pyruvate decarboxylase (PDC) starts to convert pyruvate into pH‐neutral acetaldehyde, preventing acidification of the cytosol (Kimmerer & MacDonald 1987; Harry & Kimmerer 1991). Consistent with this pyruvate overflow theory, the inhibition of mitochondrial respiration and pyruvate transport, thus increasing pyruvate concentrations in the cytoplasm, induced acetaldehyde emissions (Karl *et al.* 2002).

With the same line of reasoning, Jardine *et al.* (2012) explained the release of post‐illumination acetaldehyde, ethanol, acetic acid and acetone from mesquite branches in what they termed ‘pyruvate dehydrogenase (PDH) bypass pathway’. In mitochondria of yeast and mammals, PDH catalyses the formation of acetyl coenzyme A (CoA) from pyruvate (Wei *et al.* 2009 and references therein). In the PDH bypass mechanism, acetaldehyde is converted into acetate, which can serve as substrate for acetyl CoA synthesis in the chloroplasts. Acetyl CoA is a building block of fatty acids (Boubekeur *et al.* 2001, Wei *et al.* 2009), and together with the GLV Z‐3‐hexenol, it can form Z‐3‐hexenyl acetate in a reaction catalysed by an acyl transferase (D'Auria *et al.* 2007). Consistently, in ^13^CO_2_ labelling experiments with cottonwood (Karl *et al.* 2002), mesquite branches (Jardine *et al.* 2012) and grey poplar (Ghirardo *et al.* 2011), it was found that post‐illumination acetaldehyde partially (60%, 40% and 30%, respectively) originates from recently assimilated carbon. In mesquite branches, this was also the case for post‐illumination acetic acid, acetone, ethanol and the acetate moiety of Z‐3‐hexenyl acetate (Jardine *et al.* 2012).

Graus *et al.* (2004) speculated that acetaldehyde emissions after fast light‐to‐dark transitions are not directly related to pyruvate, but rather to acetyl CoA. Similar to Jardine *et al.* (2012) they observed enhanced acetaldehyde PIBs when poplar leaves were kept under anoxic conditions. These enhanced bursts were interpreted as acetaldehyde released from excess acetyl CoA in the case of missing hexenal from the LOX pathway to form hexenyl acetate. In their experiments, acetaldehyde PIBs were absent when the light was switched on again before the burst normally appeared. Switching on and off light repeatedly within sub‐minutes time spans, thus mimicking so‐called light flecks in a natural environment, did not result in any PIBs at all.

Carbon isotope analysis of acetaldehyde emitted from leaves (of poplar, white oak, red maple and sassafras) after mechanical stress led Jardine *et al.* (2009) to the conclusion that acetaldehyde is produced by fatty acid peroxidation reactions initiated by the accumulation of reactive oxygen species (ROS). Because of a kinetic isotope effect in the acetyl CoA formation from pyruvate by PDH, acetyl CoA‐derived products like said fatty acids are depleted in ^13^C (Park & Epstein 1961; DeNiro & Epstein 1977). Jardine *et al.* (2009) argued that acetaldehyde was as depleted in ^13^C as these unsaturated lipids, while acetaldehyde derived from ethanolic fermentation, typical under anoxic conditions (e.g. after root flooding, in O_2_‐free enclosure systems), would be less depleted (Hobbie & Werner 2004). Likewise, acetaldehyde released in leaf heating experiments seems to derive from a bulk biomass precursor, as carbon isotope analysis by Keppler *et al.* (2004) demonstrated.

Similar to acetaldehyde and the GLV, also isoprene PIBs have been reported in isoprene‐emitting plant species (Monson *et al.* 1991; Li *et al.* 2011; Li & Sharkey 2013). There is now evidence to suggest that the accumulation of methylerythritol phosphate (MEP) pathway intermediates, primarily methylerythritol cyclodiphosphate (MEcDP), is responsible for the post‐illumination isoprene burst phenomenon (Li & Sharkey 2013). The conversion of MEcDP to dimethylallyl diphosphate (DMADP) and isopentenyl diphosphate (IDP), catalysed by the iron–sulphur protein hydroxymethylbutenyl diphosphate (HMBDP) synthase, is strongly dependent on the electron transport of the photosynthetic light reaction. In darkness, HMBDP synthase switches over time to NADPH as its electron donor (Seemann & Rohmer 2007), thus relieving the intermediate pool and eventually causing the isoprene PIB in naturally isoprene‐emitting plants.

The aim of the present study was to elucidate the combined effects of heat and drought stress and elevated CO_2_ concentration on the different post‐illumination emissions of VOCs from IE and NE poplar leaves. In NE plants, the knockdown of isoprene synthase (Behnke *et al.* 2007) affects early metabolites of the plastidic isoprenoid pathway (Ghirardo *et al.* 2014) and lipid substrates of the LOX enzymes (Velikova *et al.* 2015). We used the combination of different biochemical and isoprene emission capabilities of IE and NE plants, in conjunction with the different stress‐induced responses, to deepen our understanding of the biochemical mechanisms responsible for the post‐illumination burst of acetaldehyde and GLVs. Moreover, we tested the hypothesis that the amount of VOCs released in form of PIBs provides another indicator on the plant stress status and related biochemical phenomena in the plant.

## Material and Methods

### Plant material and climate change scenarios

A detailed description of the plant material and growth conditions of the plants used in this analysis is given in Vanzo *et al.* (2015); therefore, only a brief summary is given here.

Four different genotypes of *Populus* x *canescens* (Aiton.) Sm. (INRA clone 7171‐B4; syn. *Populus tremula* x *Populus alba*) were investigated: two isoprene‐emitting lines [one wild‐type (WT) and one PcISPS:GUS/GFP line in which the PcISPS (*P.* x *canescens* isoprene synthase) promotor was fused to the *β*‐glucuronidase (GUS) and green fluorescence protein (GFP) reporter genes (for details, see Cinege *et al.* 2009)] and two well‐characterized non‐isoprene‐emitting lines [35S::PcISPS‐RNAi lines RA1 and RA2 (Behnke *et al.* 2007; Way *et al.* 2013)].

The plants were grown in four walk‐in‐size phytotron chambers at the phytotron facility of the Helmholtz Zentrum München. Each chamber contained four sub‐chambers, where plants of one genotype (WT, PcISPS:GUS/GFP, RA1 or RA2) were grown (Supporting Information Fig. S1a). In each of the chambers, a different environmental scenario was simulated: present and future controls (daily maximum temperature of 27 °C, no stress episodes) and two stress scenarios with periodic and chronic exposure to increased temperatures (T = control temperature +6 °C, daily maximum temperature of 33 °C) and water limitation (see next paragraph). The scenarios are termed as follows:

1. AC: control with ambient [CO_2_] = 380 *μ*L L^−1^.
2. EC: control with elevated [CO_2_] = 500 *μ*L L^−1^.
3. PS: periodic stress containing three cycles (each 6 days) with increased temperature and concomitant, acute drought (hereafter referred as heat and drought spell, HDS). The start of the first HDS is termed as day 1 (d1) of the experiment. Between the first and second and the second and third HDS, a recovery time of 2 days was implemented, where temperature declined to control level (27 °C) and plants were irrigated to pot capacity.
4. CS: chronic stress with slowly developing drought progressing over 22 days from d0 to d22 (during these days, temperature was increased as in PS).

The HDS in the CS and PS scenarios were followed by a final recovery time of 7 days (from d22 to d29) where temperature decreased to control level and pots were irrigated to saturation. These scenarios were considered separately and termed:

CSr: recovery from CS scenario.
PSr: recovery from PS scenario.

In the CS/CSr and PS/PSr scenarios, the ambient CO_2_ concentration was the same as in EC ([CO_2_] = 500 *μ*L L^−1^).

In our analysis, the EC scenario is the direct control of the CS/CSr and PS/PSr scenarios. There was no attempt to separate temperature and drought factors in this study.

### Plant irrigation and simulation of water scarcity

The controlled water regime was obtained using automated drip irrigation systems placed in each pot half way between the stem and the edge of the pot. Plants were exposed to the short‐term (in PS) and long‐term drought (in CS) by reducing the amount of irrigation water gradually during each HDS. In the PS scenario, three drought cycles were imposed to mimic natural wet–dry cycles in the field. In the first, second and third cycles, the amount of water was reduced by 50%, 60% and 70% compared with AC and EC, respectively. To slow down the progression of drought in the CS scenario, in the first 5 days, the irrigation amount was reduced by only 30% compared with fully watered controls in AC and EC. Every 5 days, the water amount in CS was reduced by 10% reaching a reduction of 70% compared with the controls.

### Leaf‐level gas exchange measurements and volatile organic compound analysis

Leaf‐level gas exchange measurements were made from d19 to d22 (during HDS) and d25 to d28 (during recovery from HDS). The measurements were performed using two Walz GFS‐3000 leaf cuvette systems (8 cm^2^ clip‐on‐type, Heinz Walz GmbH, Effeltrich, Germany) run in parallel. The GFS‐3000 systems logged leaf temperatures, pressures, flows, [CO_2_], [H_2_O] and plant physiological parameters [assimilation rate (A) and transpiration rate (E) were calculated automatically]. The outlets of the leaf cuvettes were passed by a heated line (*T* = 40 °C) to a proton transfer reaction time‐of‐flight mass spectrometer (PTR‐ToF‐MS, Graus *et al.* 2010); see Supporting Information Fig. S1b.

The measurements were performed in total on 256 attached leaves (leaf no. 9 counting from the apex) under standard conditions (temperature of 30 °C, photosynthetically active radiation PAR = 1000 *μ*mol m^−2^ s^−1^, air humidity of 10 000 ppmv). The cuvette was flushed with synthetic air with growth [CO_2_] (AC: 380 *μ*L L^−1^; else: 500 *μ*L L^−1^). Each measurement took 40 min, split into three time ranges: (1) 10 min leaf measurement under light conditions, (2) 20–25 min under dark conditions and (3) 5–10 min background measurement of the empty cuvette without leaf and light (blank for the PTR‐ToF‐MS). While sampling from one cuvette, a plant for the subsequent measurement could be installed in the other cuvette and was allowed to acclimatize for 40 min before the measurement began.

The PTR‐ToF‐MS was operated under standard conditions, 60 °C drift tube temperature, 540 V drift voltage and 2.3 mbar drift pressure, corresponding to an *E*/*N* of 120 Td (*E* being the electric field strength and *N* the gas number density; 1 Td = 10^−17^ V cm^2^). The instrument was calibrated once a week by dynamic dilution of VOCs using a standard gas mixture (Apel Riemer Environmental Inc., Broomfield (CO), USA), containing 20 compounds of different functionality distributed over the mass range of 30–204 amu. Limits of detection were as low as 35 pptv. Full PTR‐ToF‐MS mass spectra were recorded up to mass to charge ratio m/z 315 with a 1 s time resolution. Raw data analysis was performed using the routines and methods described elsewhere (Müller *et al.* 2013 and references therein).

In PTR‐ToF‐MS, acetaldehyde can be detected at m/z 45.034, ethanol at m/z 47.050 and isoprene at m/z 69.070. Certain substances like longer‐chain aldehydes and alcohols are prone to fragment during the ionization in the drift tube of a PTR‐MS (e.g. Fall *et al.* 1999), and therefore, the GLVs can be detected at various mass‐to‐charge ratios, for example:

- E‐2‐hexenal: m/z 99.081, m/z 81.070, m/z 57.034
- Z‐3‐hexenal: m/z 99.081, m/z 81.070
- Z‐3‐hexenol: m/z 101.097, m/z 83.086
- Z‐3‐hexenyl acetate: m/z 143.107, m/z 83.086

Post‐illumination bursts signals were background and baseline corrected and quantified by calculating the individual burst area, that is, the cumulative sum of the ion signal (given in normalized counts per second, ncps) of the burst, normalized to the single‐sided leaf area in the leaf cuvette (8 cm^2^). Additionally, slopes in the inflection point of the rising edges of PIBs (using a simple linear regression model), the time difference between switching off the light in the cuvette and the burst maxima were calculated. For methanol (m/z 33.034) and isoprene, steady‐state standard emission rates normalized to unit leaf area (nmol m^−2^ s^−1^) were calculated from the last 10 min before switching off the light.

### Enzyme extraction and lipoxygenase activity analysis

The LOX activity (EC 1.13.11.12) was measured following the protocol described in Koch *et al.* (1992). Leaf material (leaf no. 9–12 counting from the apex) of IE and NE plants was harvested at noon at the last day of stress treatment and 7 days later at the end of the recovery period. Leaves were immediately frozen in liquid N_2_ and stored at −80 °C until biochemical analyses. One‐hundred milligrammes of leaf material was homogenized with 50 mg Polyclar AT (Sigma Aldrich, Deisenhofen, Germany) on ice in 1 mL PEB (50 mM KP_i_ pH 7.0; 0.1% (v/v) Triton X‐100 and 20 mM DTT). After thoroughly mixing, the homogenates were centrifuged for 15 min at 16 000 *g*, and 0.5 mL of supernatant were loaded on a pre‐prepared NAP‐5 column equilibrated and eluted with 1 mL of KP_i_ (50 mM, pH 7.0). The sodium linoleate substrate was prepared by mixing 70 mg Tween‐20 with 70 mg linoleic acid and 4 mL H_2_O thoroughly bubbled with argon to remove oxygen. The milky solution was cleared by adding 130 *μ*L 2 N NaOH and filled up to 25 mL with oxygen‐free H_2_O. The stock solution was stored at −20 °C. The kinetic of LOX activity was immediately measured spectrophotometrically at 234 nm with a dwell time of 10 s. Two hundred microlitre of extract was mixed with 800 *μ*L KP_i_ (50 mM pH 7.0) and 8 *μ*L of substrate (linoleic acid). After stabilization of enzyme activity, the reaction kinetic δ234 nm was monitored for 5 min. LOX activity was measured at 25 °C and is reported as microkatal per milligramme total protein, assuming the molecular extinction coefficient of 2.5 × 10^4^ M^−1^ cm^−1^ for the conjugated diene product (Axelrod *et al.* 1981). For each protein extract, four technical replicates were analysed. The protein concentration was measured using the Qubit^TM^ protein assay kit (Life Technologies GmbH, Darmstadt, Germany).

### Statistical analyses

All measured data were statistically analysed in order to gain insight into the effects of plant genotype and environmental conditions on the release of PIBs. The experiment was designed in a way that all plants measured from the same sub‐chamber (cf. Supporting Information Fig. S1a) were handled as technical replicates; that is, the data were averaged and used as single biological replicate. For each of the IE, respectively, and NE genotypes (IE consisting of WT and PcISPS:GUS/GFP lines; NE consisting of RA1 and RA2 lines), we analysed leaf gas exchange and PIBs of four independent plant leaves in the CS, PS, CSr and PSr scenarios and of eight independent plant leaves in the AC and EC scenarios. The experiment was repeated under exactly the same climate and plant conditions to generate four biological replicates (sub‐chambers) per treatment (scenario) for the IE and NE genotypes, respectively. Altogether, 256 plants were surveyed.

Correlation analyses were performed using principal component analysis (PCA) and orthogonal partial least square regression (OPLS) (SIMCA‐P v13, Umetrics, Umeå, Sweden) between IE or NE genotypes, VOC PIBs area, slopes, enzyme activity and gas exchange parameters (net assimilation, dark respiration and transpiration under light and dark conditions) (all set as *X*‐variables). Thereby, established procedures to analyse MS data were followed as reported previously (Ghirardo *et al.* 2005; Ghirardo *et al.* 2012; Kreuzwieser *et al.* 2014; Vanzo *et al.* 2014; Velikova *et al.* 2014; Velikova *et al.* 2015). For OPLS analysis, seven different *Y*‐variables describing the NE genotypes, the four scenarios (PS, CS, PSr and CSr) and the two controls (AC and EC) were created. Each *Y*‐variable was set to 1 when the sample belonged to the corresponding class and to 0 otherwise. The *X*‐variables were logarithmically transformed and centred, and each type of data was blockwise scaled with 1 sd^−1^. Each calculated significant principal component was validated using ‘full cross‐validation’, with 95% confidence level on parameters. The regression model OPLS was tested for significance using CV‐ANOVA (Eriksson *et al.* 2008).

Moreover, two‐way ANOVAs were performed in SigmaPlot (v13.0, Systat Software Inc., San Jose, CA, USA) on the means of the measured parameters using the two factors ‘Scenarios’ (AC, EC, PS, CS, PSr and CSr) and ‘Genotypes’ (IE and NE). Tukey post‐hoc test followed the ANOVA to pairwise compare particular scenarios and the poplar genotypes. Results were considered significant at *P* < 0.05.

## Results and Discussion

In our experiments, we could observe PIBs of acetaldehyde, ethanol, isoprene (only in IE plants), hexenal, hexenol and hexenyl acetate isomers. From time to time, small PIBs of two unidentified compounds with sum formulas C_5_H_11_^+^ (m/z 71.086) and C_6_H_9_O_2_^+^ (m/z 113.060) were observed. C_5_H_11_^+^ might be a fragment of a carbonyl deriving from a not yet well‐established LOX pathway yielding C_5_ compounds (Salch *et al.* 1995; Fall *et al.* 2001; Jardine *et al.* 2012; Shen *et al.* 2014). C_6_H_9_O_2_^+^ could be a fragment of (E‐2)‐4‐hydroxy‐2‐hexenal, a substance that was shown to be formed out of Z‐3‐hexenal in soybean (Takamura & Gardner 1996). Because in our experiments the measured emissions of these unidentified compounds were low, they will not be considered further here.

**Figure 1:**
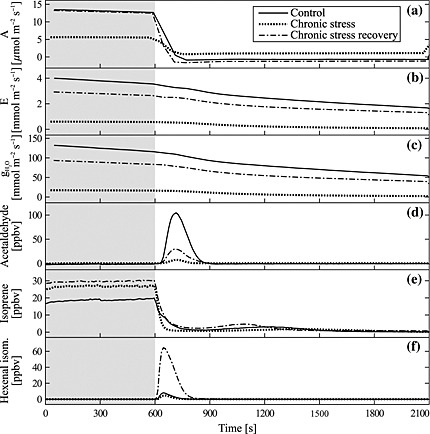
Gas exchange parameters [assimilation rate A (a), transpiration rate E (b) and water vapour conductance gH_2_O (c)] and signals of selected volatile organic compounds (VOCs) (d–f) measured in leaf cuvette experiments simulating light‐to‐dark transitions. The sample plants were grown under different climate scenarios: elevated control (solid line), chronic stress (dotted line) and chronic stress recovery (dot‐dashed line). The grey shaded area denotes a 10 min light period at the beginning of each experiment. After switching off the light, post‐illumination bursts (PIBs) of several VOCs appeared such as acetaldehyde (d), isoprene (e) and the isomers Z‐3‐hexenal and E‐2‐hexenal (f). Under chronic stress, PIBs were strongly suppressed. While PIBs of acetaldehyde were weaker in plants recovering from chronic heat and drought stress than in control plants, PIBs of green leaf volatiles like the hexenal isomers were enhanced. Signals are means of *n* = 8 (elevated control) and *n* = 4 (chronic stress and chronic stress recovery).

Figure 1 shows examples of the effect of different treatments (EC, CS and CSr) on plant physiological parameters (assimilation, transpiration and stomatal conductance) and emissions of specific VOCs from IE plants grown under elevated [CO_2_]. During HDS, most of the VOC PIBs were reduced. In the recovering phase, however, the pattern of the PIBs differed from the control treatment. In the following sections, we will show that the amount of VOC emissions following the rapid light‐to‐dark transitions depended both on the plant genotype and the growth condition. According to their emission rates in the different treatments, they could be subdivided into three different classes linked to different biochemical origins.

### Multivariate data analyses separate plant genotypes and treatment effects

**Figure 2:**
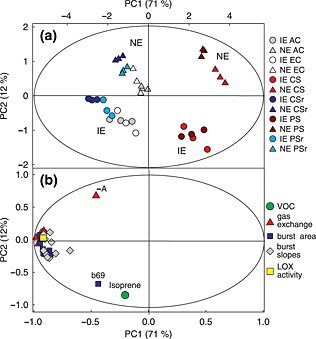
Score (a) and loading (b) plots of principal component (PC) analysis calculated using means of volatile organic compound (VOC) bursts (integral of the peak curves), their slopes (through their rising edge), leaf‐level VOC emission rates (of methanol and isoprene) during light conditions, gas exchange parameters (net assimilation, dark respiration; transpiration under light condition and transpiration under dark condition) and *in vitro* activities of lipoxygenase (LOX) enzymes data. (a) isoprene‐emitting (IE) (WT + PcISPS:GUS/GFP) circles; isoprene non‐emitting (NE) (RA1 + RA2), triangles. (b) Each parameter class is indicated by a different symbol. T2 ellipses denote a significance level of *α* = 0.05. AC, ambient control; EC, elevated control; CS, chronic stress; PS, periodic stress; CSr, chronic stress recovery; PSr, periodic stress recovery; −A, dark respiration; b69, burst area isoprene.

Multivariate data analyses revealed a clear pattern of the samples subjected to the different climate scenarios, originating from IE and NE plant genotypes (Figs 2 & S2). Except for isoprene, all PIBs appeared in plants grown under unstressed conditions (AC + EC scenario), independently from plant genotypes, but they were mostly suppressed in plants grown under CS and PS scenarios. This is illustrated in Fig. 2: the first principal component (PC1) of PCA separates CS and PS samples (right side of the loading plot in Fig. 2a) from samples grown in the AC, EC, CSr and PSr climate scenarios; VOC PIBs are located on the opposite side (negative correlation) of the loading plot (Fig. 2b). In contrast to IE plants, the NE genotypes were affected differently by the CS and PS treatments. This effect is illustrated by the clear treatment‐dependent clustering within the NE samples (Fig. 2a). The PIBs originating from samples under CS and PS decreased in concert with the physiological parameters net assimilation and transpiration rates, but also with methanol emission rates (under light) and *in vitro* LOX enzyme activities.

We employed OPLS analysis to further investigate which parameters correlate strongest with the plant genotypes or the different climatic conditions ([Link], [Link]). The distinction between the four different plant genotypes mainly originated from their ability to either emit or not emit isoprene under constant light and was independent of treatment, as indicated by the high negative correlation coefficient (Fig. S3a). The genotype effect was clearly revealed by the second principal component (PC2) in both PCA and OPLS (Figs 2 & S2, respectively) and was related to constitutive isoprene emission rates and partially to PIBs of isoprene and dark respiration (denoted as ‘IS’, ‘b69’ and ‘–A’, respectively, in both Figs 2b & S2; see also correlation coefficients in Fig. S3a). This is the reason why we pooled together WT and PcISPS:GUS/GFP genotypes (IE) as well as RA1 and RA2 plants (NE) and considered for further analysis only the IE and NE class.

Overall, the OPLS model was very reliable as seen by the high numerical value of *Q* ^2^(*Y*) = 94.4%. Other performance indicators were *R* ^2^(*X*) = 80%, *R* ^2^ = 36% and *R* ^2^(*Y*) = 67% using four PC. Furthermore, we used the CV‐ANOVA, based on cross‐validated predictive residuals (Eriksson *et al.* 2008) to test the significance of the OPLS models created and found that the model was highly significant to describe genotype and treatments, resulting in the following *P*‐values: <1e^−14^ (describing NE/IE genotypes), 4.9e^−9^ (AC), 2.0e^−7^ (EC), 2.7e^−13^ (PS), 2.7e^−14^ (CS) and 3.9e^−10^ (CSr). Only the model explaining samples of the PSr scenario was not found significant (*P* = 0.36).

### Heat and drought spells had only little effect on post‐illumination bursts of isoprene

In poplar plants, isoprene is the most abundant VOC emitted from leaves; also, other VOCs can be emitted significantly in a constitutive manner with leaf age‐dependent emission potentials (Ghirardo *et al.* 2011). In the transgenic poplar plants used in our experiments, knocking down of isoprene synthase (ISPS) causes the almost complete suppression of isoprene biosynthesis (Behnke *et al.* 2007). This had also impact on the PIBs of isoprene.

**Figure 3:**
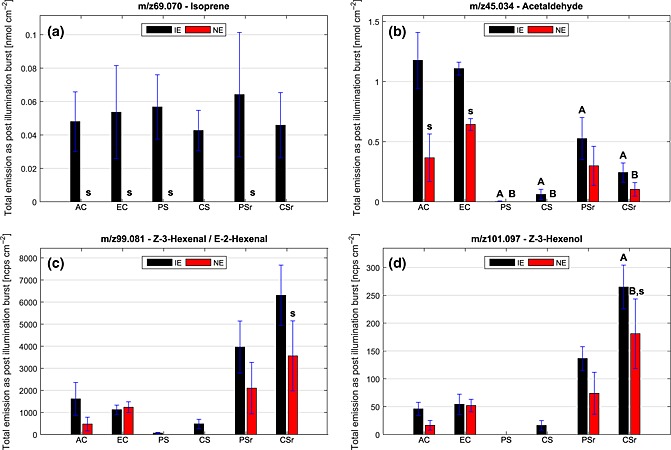
Mean total emissions (±SE) of isoprene (a) and selected oxygenated volatile organic compounds (VOCs) (b–d) in form of post‐illumination bursts (PIBs). In the PS and CS scenarios, all PIBs except those of isoprene were strongly suppressed. Isoprene non‐emitting (NE) species generally showed less intense PIBs of oxygenated VOC than isoprene‐emitting (IE) species. PIBs of the C_2_ compounds like acetaldehyde were less intense in the stress recovery treatments (PSr and CSr) than in the corresponding control treatment (EC). Contrary, PIB of C_6_ compounds like hexenal and hexenol were enhanced in plants recovering from heat and drought spell. Letters denote statistically significant (*P* < 0.05) different values: (s) between IE and NE plants within the climate scenario, (A) in regard to the control treatment (EC) within IE plants and (B) in regard to the control treatment within NE plants (cf. Supporting Information Table S1). AC, ambient control; EC, elevated control; CS, chronic stress; PS, periodic stress; CSr, chronic stress recovery; PSr, periodic stress recovery.

In experiments with IE plants, switching off the light caused the well‐known PIBs of isoprene (cf. Fig. 1). During these bursts, approximately 0.04 to 0.06 nmol cm^−2^ of isoprene was emitted, independently from the plant treatment (Fig. 3a; *P* > 0.05; Supporting Information Table S1). Only in the CS scenario was the absolute amount of isoprene emitted as PIB slightly lower, although not statistically different. The observation that the PIB isoprene emission, like constitutive isoprene emission, is unaffected by HDS, supports the current knowledge that in both cases the emitted isoprene molecules derive from the same substrate. As mentioned earlier, the PIB of isoprene originates from a pool of MEP pathway intermediates after the light‐dependent HMBDP synthase enzyme has switched to NADPH as electron donor, and newly available DMADP is quickly converted into isoprene by ISPS (Li & Sharkey 2013).

**Figure 4:**
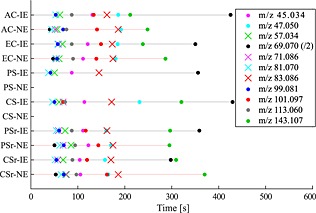
Mean time delays (expressed in seconds) after which the individual PIB maxima appeared after light‐to‐dark transitions (*t* = 0 s) in the different climate scenarios. Solid circles represent parent ions, while crosses represent known fragments. Values of m/z 69.070 were divided by 2. IE, isoprene‐emitting; NE, isoprene non‐emitting; AC, ambient control; EC, elevated control; CS, chronic stress; PS, periodic stress; CSr, chronic stress recovery; PSr, periodic stress recovery.

Consistently, the lack of ISPS in NE plants resulted in a suppression of the typical isoprene PIBs, although in NE plants, the chloroplastic DMADP pool is much higher than in IE plants (Ghirardo *et al.* 2014). However, from time to time, we detected some tiny PIBs at the mass‐to‐charge ratio of isoprene (m/z 69.070) in NE plants recovering from HDS. It is unclear whether these signals were caused by isoprene or by a fragment of some interfering C_5_ compound (Salch *et al.* 1995; Fall *et al.* 2001; Jardine *et al.* 2012; Shen *et al.* 2014). The latter could be supported by an apparent correlation of the appearance of these bursts with PIBs of an unidentified C_5_ compound at m/z 71.086 about half a minute earlier. If these occasional early burst signals at m/z 69.070 appeared also in IE plants, they could likely not be resolved from the high isoprene signals slowly decaying after quitting the leaf illumination (cf. Fig. 1). While the maximum of regular isoprene PIBs appeared after a mean time span of 729 s (minimum ~ 400 s, maximum ~ 1294 s), the PIBs of NE plants appeared much faster after 102 s (minimum ~ 60 s, maximum ~ 193 s); see Fig. 4. Because of these facts, in such cases, the PIB values were neglected in the actual analysis of isoprene PIBs (and Fig. 3a).

### Acetaldehyde post‐illumination bursts as a result of DXS activity?

Acetaldehyde emissions following light‐to‐dark transitions have been observed in several plant species (e.g. Holzinger *et al.* 2000; Karl *et al.* 2002; Graus *et al.* 2004; Ghirardo *et al.* 2011; Jardine *et al.* 2012). In *Quercus Ilex* L*.* the PIBs of acetaldehyde were accompanied by ethanol PIBs (Holzinger *et al.* 2000), while in sycamore, aspen, cottonwood and maple leaves, no concomitant ethanol emissions were observed (Karl *et al.* 2002). Jardine *et al.* (2012) detected also PIBs of acetic acid and acetone, which were thought to derive from the same synthetic pathway (see also Jardine *et al.* 2010). This variation in the pattern of C_2_ compound emissions is indicative for a high complexity of the involved synthesis pathways and the impact of the plants' biochemical status on the emission.

In our experiments, the absolute amounts of C_2_ compound (acetaldehyde + ethanol) emissions during PIBs were lower in NE than in IE plants (*P* < 0.001) and they were almost totally suppressed in the CS and PS scenarios (cf. Table 1 and Fig. 3b). This is also depicted by the negative OPLS correlation coefficients of acetaldehyde and ethanol in these scenarios (Fig. S3d,e). In plants recovering from HDS, the emissions as PIBs of the C_2_ compounds were significantly lower than in the corresponding control treatments (*P* < 0.007 for acetaldehyde, *P* < 0.09 for ethanol).

**Table 1:** Genotype and climate scenario specific post‐illumination volatile organic compound emissions in poplar

Genotype	Scenario	m/z 45	m/z 47	m/z 57	m/z 69	m/z 71	m/z 81	m/z 83	m/z 99	m/z 101	m/z 113	m/z 143
IE	AC	26 842 ± 5 345	218 ± 46	154 ± 76	681 ± 259	45 ± 21	3 058 ± 1 422	2 364 ± 436	1 616 ± 743	46 ± 12	9 ± 5	66 ± 11
NE	AC	8 459 ± 4 639	82 ± 36	44 ± 26	0 ± 0	12 ± 9	918 ± 607	1 213 ± 511	474 ± 309	17 ± 9	2 ± 2	33 ± 13
IE	EC	25 195 ± 1 318	246 ± 31	124 ± 45	750 ± 385	27 ± 4	2 034 ± 319	3266 ± 881	1 124 ± 207	54 ± 18	3 ± 1	87 ± 27
NE	EC	14 635 ± 1 193	140 ± 14	122 ± 30	0 ± 0	28 ± 6	2 324 ± 438	2 968 ± 611	1 237 ± 244	52 ± 11	3 ± 1	93 ± 16
IE	PS	33 ± 33	0 ± 0	12 ± 7	803 ± 278	0 ± 0	114 ± 56	262 ± 129	66 ± 31	0 ± 0	0 ± 0	0 ± 0
NE	PS	0 ± 0	0 ± 0	0 ± 0	0 ± 0	0 ± 0	0 ± 0	0 ± 0	0 ± 0	0 ± 0	0 ± 0	0 ± 0
IE	CS	1 423 ± 1 004	14 ± 9	62 ± 16	605 ± 176	8 ± 8	878 ± 449	1 332 ± 346	481 ± 209	16 ± 9	1 ± 1	14 ± 14
NE	CS	0 ± 0	0 ± 0	0 ± 0	0 ± 0	0 ± 0	0 ± 0	0 ± 0	0 ± 0	0 ± 0	0 ± 0	0 ± 0
IE	PSr	12 090 ± 4 163	156 ± 27	948 ± 315	904 ± 525	93 ± 40	5 913 ± 2 322	5 696 ± 958	3 960 ± 1 180	136 ± 21	13 ± 8	112 ± 6
NE	PSr	6 965 ± 3 802	83 ± 49	364 ± 202	0 ± 0	57 ± 33	3 597 ± 2 029	3 830 ± 1 721	2 100 ± 1 167	74 ± 38	11 ± 6	83 ± 36
IE	CSr	5 606 ± 1 897	79 ± 23	874 ± 159	644 ± 272	181 ± 51	11 048 ± 2 911	10 296 ± 543	6 304 ± 1 368	265 ± 39	25 ± 11	210 ± 25
NE	CSr	2 405 ± 1 318	36 ± 21	393 ± 161	0 ± 0	107 ± 55	6 747 ± 3 182	9 200 ± 3 177	3 561 ± 1 585	181 ± 62	17 ± 10	187 ± 49

Values are means of *n* = 4 biological replicates (±SE) and denote the area under the post‐illumination bursts, normalized to the leaf area. Because the proton transfer reaction time‐of‐flight mass spectrometer has no isomeric separation capability and signals at several mass‐to‐charge ratios (m/z) correspond to fragments of more than one GLV, assigning sensitivities to certain ion signals is difficult. The emissions are therefore given in normalized counts per second per squared centimetre leaf area [ncps cm^−2^_Leaf area_]. Refer to the main text for the assignment of molecules to m/z ratios. Statistical results are given in Supporting Information Table S1.

IE, isoprene‐emitting; NE, isoprene non‐emitting; AC, ambient control; EC, elevated control; CS, chronic stress; PS, periodic stress; CSr, chronic stress recovery; PSr, periodic stress recovery.

Three sources of post‐illumination acetaldehyde emission can be imagined: (1) reaction of ADH to convert ethanol to acetaldehyde, (2) action of pyruvate decarboxylase in the cytosol and (3) release during the complex kinetics of deoxyxylulose phosphate synthase.

The peaks of the ethanol PIBs appeared always after those of acetaldehyde (mean time difference of burst peaks: 58 ± 18 s; Fig. 4). The obvious correlation (*R* ^2^ = 0.98) between acetaldehyde and ethanol PIBs (cf. Table 1) rather supports the reverse reaction of the ADH, in which ethanol is formed out of acetaldehyde.

When the light is abruptly turned off, CO_2_ will continue to be fixed until all of the ribulose‐1,5‐bisphosphate (RuBP) in the Calvin–Benson–Bassham (CBB) cycle is consumed (Laisk *et al.* 1984). However, reducing power in the form of NADPH is not available, resulting in a large increase in phosphoglyceric acid (PGA) and pyruvate immediately after a light to dark transient. This pyruvate could be consumed by cytosolic PDC and form acetaldehyde in what Karl *et al.* (2002) called the ‘pyruvate overflow mechanism’ and Jardine *et al.* (2012) the ‘PDH bypass pathway’.

The third potential source of acetaldehyde is 1‐deoxy‐D‐xylulose‐5‐phosphate synthase (DXS). DXS is responsible for the formation of 1‐deoxy‐D‐xylulose‐5‐phosphate (DXP) from pyruvate and glyceraldehyde‐3‐phosphate (GAP) in the first step of the MEP pathway (Ghirardo *et al.* 2010). DXS first forms a decarboxylated thiamine diphosphate (ThDP)–pyruvate complex to make hydroxyethyl ThDP. This step is the same as the first step of PDC. In the case of PDC, the hydroxyethyl ThDP is hydrolysed to acetaldehyde and ThDP. It is worth future research into whether the post‐illumination stromal environment causes DXS to act as a PDC for a short time.

Another possibility is that DXS could reverse part of the reaction sequence to liberate GAP and hydroxyethyl‐ThDP or just acetaldehyde. Release of GAP from DMADP has been observed (A. Banerjee, T.D. Sharkey, unpublished).

This proposed mechanism is coherent with findings of Karl *et al.* (2002). In ^13^CO_2_ labelling experiments with cottonwood leaves, after about 100 min of ^13^CO_2_ fumigation in light, they found comparable labelling of both isoprene (78%) and acetaldehyde (60%).

Previous observations of enhanced acetaldehyde emissions when using PDH (Graus *et al.* 2004) or pyruvate transport inhibitors (Karl *et al.* 2002) and the observed dependence of the acetaldehyde PIBs on CO_2_ availability and assimilation rates (Jardine *et al.* 2012) are consistent with the proposed role of DXS in the acetaldehyde formation involving pyruvate too. Eventually, acetaldehyde can be further processed by ALDH to acetic acid, which has been observed following PIBs of acetaldehyde (Jardine *et al.* 2012).

A role for DXS in the acetaldehyde burst is also consistent with data presented in Figs 3 and S3a, showing lower acetaldehyde PIBs in NE than in IE plants. It is known that high DMADP levels in chloroplasts act as feedback regulator of the MEP pathway, by inhibiting the activities of DXS (Wolfertz *et al.* 2004; Banerjee *et al.* 2013; Ghirardo *et al.* 2014). In NE plants, the suppression of ISPS leads to an accumulation of chloroplastic DMADP, which causes a strong down‐regulation of the DXS activity (Ghirardo *et al.* 2014) and DXS protein levels, resulting in a reduced metabolic C‐flux throughout the MEP pathway. Taken together, the lower DXS activities and protein levels of NE compared with that of IE plants (Ghirardo *et al.* 2014) and the proposed change in DXS catalytic properties might be the reason of the low PIBs of acetaldehyde from NE leaves. Moreover, the MEP pathway is ubiquitous in all plant species, which explains why the PIB emissions of acetaldehyde are detected independently from the trait to emit isoprene (Brilli *et al.* 2011).

Nonetheless, we cannot exclude that the differences between IE and NE plants derive from comprehensive changes in metabolite concentrations (Way *et al.* 2013) and metabolic fluxes (Ghirardo *et al.* 2014), rearrangements of enzymatic and functional protein contents (Velikova *et al.* 2014) or changes in lipid composition (Velikova *et al.* 2015). Moreover, this proposed explanation for the differences between acetaldehyde emissions of IE and NE genotypes does not fully explain why the PIBs were suppressed in drought and temperature stressed plants (CS and PS scenarios), although the constitutive emission of isoprene, an end product in the MEP pathway, in IE plants is unaffected by HDS. A possible explanation for this effect can be an up‐regulation of ADH and ALDH genes during HDS, as has been reported for Arabidopsis (Seki *et al.* 2002).

### Reduced lipoxygenase activity in HDS‐stressed plants suppressed PIBs of green leaf volatiles

In unstressed plants (AC and EC) and plants recovering from HDS (CSr and PSr), switching off the light caused a strong transient release of different volatile C_6_ compounds (GLVs). The GLVs could be associated with their precursor *α*‐linolenic acid (C18:3), the most abundant fatty acid in lipids composing the chloroplast membranes of grey poplar (Velikova *et al.* 2015). This fact allowed to infer the isomeric structure of the GLVs emitted.

As examples for GLV emissions, we show the amount of the isomers E‐2‐hexenal/Z‐3‐hexenal and Z‐3‐hexenol emitted as PIBs under the different climate scenarios (Fig. 3c,d). Generally, the PIBs of the C_6_ compounds showed behaviour similar to the PIB emissions of the C_2_ compounds in terms of the [CO_2_] and HDS effect. Elevated growth [CO_2_] influenced the PIBs of GLVs in NE plants only, when compared with AC (Fig. 3c,d). Overall, PIBs of GLVs negatively correlated to NE plants (Fig. S3a). Apart from the EC scenario, NE genotypes generally showed lower PIBs of GLVs (cf. Table 1 and Fig. 3) compared with IE genotypes.

**Figure 5:**
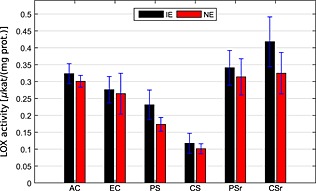
Mean activity of lipoxygenase (LOX) enzymes (±SE) in isoprene‐emitting (IE) and isoprene non‐emitting (NE) poplar plants in the different climate scenarios. In the chronic and periodic stress scenarios (CS and PS), the LOX activity was markedly reduced. Results of the statistical analysis are reported in Supporting Information Table S2. AC, ambient control; EC, elevated control; CSr, chronic stress recovery; PSr, periodic stress recovery.

Additional biochemical analyses revealed that the *in vitro* LOX protein activities were reduced in protein extracts from NE compared with IE (Figs 5 & S3b) and correlated well with NE plants (Fig. S3a). Thus, the lower amount of GLVs released from NE genotypes during light‐to‐dark transitions appears to be a consequence of both the reduced substrate availability (*α*‐linolenic acid for the LOX enzymes; Velikova *et al.* 2015) and the lower LOX enzyme activities.

The PIBs of GLVs were almost completely suppressed during HDS (cf. Fig. 3), independently from genotype. Previous studies have shown rather an increase in GLV emissions during heat stress, although not in the course of light‐to‐dark transitions (Gouinguené & Turlings 2002; Loreto *et al.* 2006; Hartikainen *et al.* 2009; Behnke *et al.* 2013). Under light, neither IE nor NE plants showed any GLV emissions under the PS and CS treatments (Vanzo *et al.* 2015, supplemental data), indicating that the stress exposure was not detrimental for membranes. The applied eustress is per definition a mild, stimulating stress, strengthening plant resistance (Lichtenthaler 1996). It can be speculated that the stress plants experienced in the studies cited in the previous text exceeded the plant's limit of tolerance (=resistance minimum), leading to membrane damage and consequently GLV emissions.

In our experiments, the suppressed GLV PIBs correlated with a notable reduction of *in vitro* LOX activities found from leaf extracts (Figs 2b & 5), which was more pronounced in chronically stressed plants than in periodically stressed ones. However, even in stressed plants, the apparent LOX activity did not drop to zero, indicating that additional mechanisms might have been involved in the suppression of *in vivo* LOX activity following the light‐to‐dark transition. Earlier studies revealed altered portions of saturated and unsaturated fatty acids in response to drought. For example, drought stress decreased the ratio of *α*‐linolenic (C18:3) to linoleic acid (C18:2) in *Carthamus tinctorius* (Hamrouni *et al.* 2001) and caused a reduction in the abundance of C18:3 fatty acids as well as a complete disappearance of palmitoleic acid (C16:1) in *Salvia officinalis* leaves (Bettaieb *et al.* 2009). In addition, in Bermuda grass (*Cynodon dactylon*), the portion of unsaturated fatty acids decreased during drought stress (Zhong *et al.* 2011). It is likely that the reduction in unsaturated fatty acids in these examples also would cause a reduction of the PIBs of GLVs when analysed during drought stress. Another possible explanation for reduced GLV PIBs in HDS plants is the effect of elevated abscisic acid (ABA) levels. Drought stress leads to increased ABA transport from the roots to the leaves (e.g. Wilkinson & Davies 2002). It has been shown that ABA has a strong inhibitory effect on LOX, while it is increasing the antioxidant capacity in *Orthosiphon stamineus* (Ibrahim & Jaafar 2013). Both effects might result in a reduced formation of GLVs as we have seen herein. The strong suppression of GLV, but also acetaldehyde PIBs under drought and temperature stress, may therefore be used as non‐invasive marker for plant phenotyping (Niederbacher *et al.* 2015).

Apparently, in our experiments, the HDS had no remarkable effect on the reaction speed during the formation of the LOX products (Fig. 4). Only the end product Z‐3‐hexenyl acetate seems to have been formed with a certain delay in CS/CSr and PS/PSr plants compared with control plants.

A remarkable difference between the PIBs of GLVs and acetaldehyde/ethanol became evident in plants recovering from HDS (CSr and PSr scenarios). The PIBs of GLVs were markedly more pronounced in CSr and PSr plants compared with control plants, while the PIB emissions of C_2_ compounds were reduced (Table 1 and see previous section). This indicates that both types of PIBs were biochemically independent, either deriving from different substrate pools or requiring different enzymatic formation processes (e.g. Graus *et al.* 2004; Jardine *et al.* 2012). Moreover, the differences in the PIBs of acetaldehyde and Z‐3‐hexenyl acetate (reduced vs. enhanced emissions) in the CSr and PSr scenarios indicate that these are not directly linked wire acetyl CoA, unlike Jardine *et al.* (2012) concluded from their ^13^C labelling experiments.

We hypothesize that enhanced GLV PIBs after recovery might have been a result of altered membrane composition during HDS and an increase of LOX activity (Fig. 5 and Supporting Information Table S2). During stress events, plants remodel membrane fluidity (see previous text) and also release large amounts of *α*‐linolenic acid from membrane lipids (Upchurch 2008). In plants recovering from HDS, ABA levels are recovering to normal values faster than those of the free lipids. Furthermore, the fatty acid composition might change back to more unsaturated ones. Consequently, in CSr and PSr plants, higher enzyme activity of 13‐LOX, most obvious of IE plants (Fig. 5), and substrate pools for the GLV formation were available. These facts eventually resulted in higher GLV PIBs in plants recovering from HDS.

## Conclusion

Our experiments showed that PIBs of acetaldehyde, ethanol and GLVs are potential markers for the heat and drought stress status of poplar plants. Under physiological stress conditions, these PIBs almost disappeared. While PIBs of acetaldehyde were lower in plants recovering from HDS in respect to the corresponding control plants, PIBs of GLVs were enhanced. This observation supports the suggestion that acetaldehyde and GLV PIBs are not directly linked biochemically.

Reduced acetaldehyde PIBs in NE plants were potentially a result of the feedback inhibition of DXS in the MEP pathway in plants lacking ISPS. Analysing the kinetic properties of DXS to release acetaldehyde from hydroxyethyl‐ThDP under biochemical conditions experienced just after light‐to‐dark transition might help clarifying the origin of the PIB acetaldehyde emissions in plants.

Our results demonstrated that in grey poplar, analysing PIBs of GLVs might be useful as a non‐invasive phenotypic marker of stress‐induced changes in free fatty acids or fatty acid composition of chloroplast membranes and *in planta* LOX activity under given environmental conditions. Based on the present data, it might be promising to test in other plant species whether the reduction of PIBs of C_2_ compounds and GLVs is a general phenomenon and is applicable as a non‐invasive phenotypic marker, quantifying the susceptibility of genotypes under drought and heat (eu)stress.

## Conflict of Interest

The authors have no conflicts of interest to declare.

## Supporting information




**Figure S1.** Schematic of the plant chamber arrangement (a) and the setup used for the gas exchange measurements (b). The four different plant genotypes (WT, PcISPS:GUS/GFP, RA1, RA2) were grown in four individual sub‐chambers within four phytotron chambers at the Helmholtz Zentrum München (a). In each chamber a different climate scenario, applying either no stress or heat and drought stress under specific CO_2_ concentrations, was simulated. In the leaf gas exchange measurements (b), a Walz‐GFS 3000 leaf cuvette system (consisting of control unit 3100‐C and standard leaf cuvette 3010‐S) was used to control and monitor temperature, pressure, air humidity and CO_2_. The PTR‐ToF‐MS sampled from the leaf cuvette back stream line and could be switched to sample from either gas exchange system.
**Figure S2.** Score (a) and correlation scaled loading (b) plots of Orthogonal Partial Least Squares (OPLS) calculated using VOC bursts (integral of the peak curves), their slopes (through their rising edge), leaf‐level VOC emission rates (of methanol and isoprene) during light conditions, gas‐exchange parameters and *in‐vitro* activities of LOX enzymes data. (a) IE (WT + PcISPS:GUS/GFP) circles; NE (RA1 + RA2) triangles; each scenario is indicated by a different colour. Each point denotes an individual sample. (b) *Y* ‐variables are indicated by black squares; each parameter class is indicated by a different symbol. Inner, middle and outer ellipses denote the 50, 75, 100% limits, respectively. OPLS model fits: *Q* ^2^(*Y* ) = 94.4%; *R* ^2^(*X*) = 80%, *R* ^2^ = 36%, *R* ^2^(*Y* ) = 67% (using four PC). Abbreviations used: IE: isoprene emitter, NE: non isoprene emitter, AC: ambient control, EC: elevated control, CS: chronic stress, PS: periodic stress, CSr: chronic stress recovery, PSr: periodic stress recovery, *A*: net assimilation, −*A*: dark respiration, *Eon*: transpiration under light conditions, *E* *Eoff*: transpiration under dark conditions, *Met*: methanol, *IS*: isoprene, *b*69: burst area isoprene.
**Figure S3.** Correlation coefficient plots of Orthogonal Partial Least Squares (OPLS) related to respective model class information: (a) plant genotype, (b) AC, (c) EC, (d) PS, (e) CS, (f) CSr. Parameter colours follow the plot legend in Fig. S2. *P*‐values (CV‐ANOVA): (a) < 1*e* ^−14^, (b) 4.9*e* ^−9^, (c) 2.0*e* ^−7^, (d) 2.7*e* ^−13^, (e) 2.7*e* ^−14^, (f) 3.9*e* ^−10^. Correlation coeffcients of the PSr class were not included because the corresponding OPLS model had no signicance (P = 0.36). Abbreviations used: A: net assimilation, ‐*A*: dark respiration, *Eon*: transpiration under light conditions, *Eoff*: transpiration under dark conditions, *Met*: methanol, *IS*: isoprene, *s*: burst slopes, *b*: burst area. Numbers correspond to the nominal mass of the detected ions, e.g. 45: acetaldehyde; 47: ethanol.
**Table S1.** P‐values obtained from two‐way ANOVAs and Tukey post‐hoc tests for the area under the post illumination bursts detected at different mass‐to‐charge ratios (*m*/*z*). Signifficant differences are marked in bold when *P* < 0.05.
**Table S2.** P‐values obtained from two‐way ANOVAs and Tukey post‐hoc tests for the LOX activity in different plant genotypes grown under the various climate scenarios. Significant differences are marked in bold when *P* < 0.05.

Supporting info item

Supporting info item

Supporting info item

Supporting info item

Supporting info item
