# Supplementary material for: Effects of heat and drought stress on post‐illumination bursts of volatile organic compounds in isoprene‐emitting and non‐emitting poplar
Source: Plant Cell Environ. 2016 Jan 18;39(6):1204–15. doi: 10.1111/pce.12643 (PMC4982041; doi:10.1111/pce.12643)
Supplement: Supplementary file 1 — Supporting info item [file PCE-39-1204-s001.pdf]

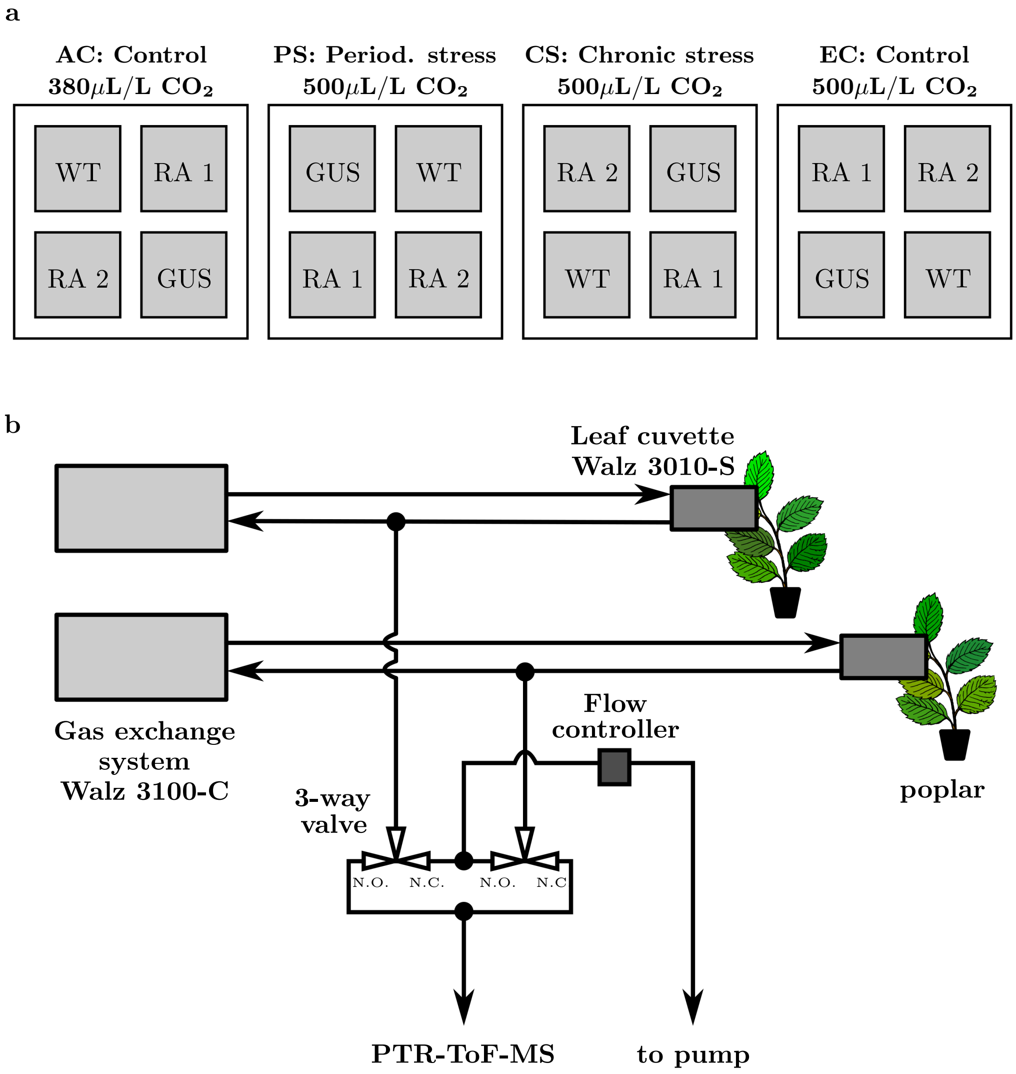

Figure S1: Schematic of the plant chamber arrangement (**a**) and the setup used for the gas exchange measurements (**b**). The four different plant genotypes (WT, PcISPS:GUS/GFP, RA1, RA2) were grown in four individual sub-chambers within four phytotron chambers at the Helmholtz Zentrum München (**a**). In each chamber a different climate scenario, applying either no stress or heat and drought stress under specific CO_2 concentrations, was simulated. In the leaf gas exchange measurements (**b**), a Walz-GFS 3000 leaf cuvette system (consisting of control unit 3100-C and standard leaf cuvette 3010-S) was used to control and monitor temperature, pressure, air humidity and CO_2. The PTR-ToF-MS sampled from the leaf cuvette back stream line and could be switched to sample from either gas exchange system.
