# Supplementary material for: Effects of heat and drought stress on post‐illumination bursts of volatile organic compounds in isoprene‐emitting and non‐emitting poplar
Source: Plant Cell Environ. 2016 Jan 18;39(6):1204–15. doi: 10.1111/pce.12643 (PMC4982041; doi:10.1111/pce.12643)
Supplement: Supplementary file 2 — Supporting info item [file PCE-39-1204-s002.pdf]

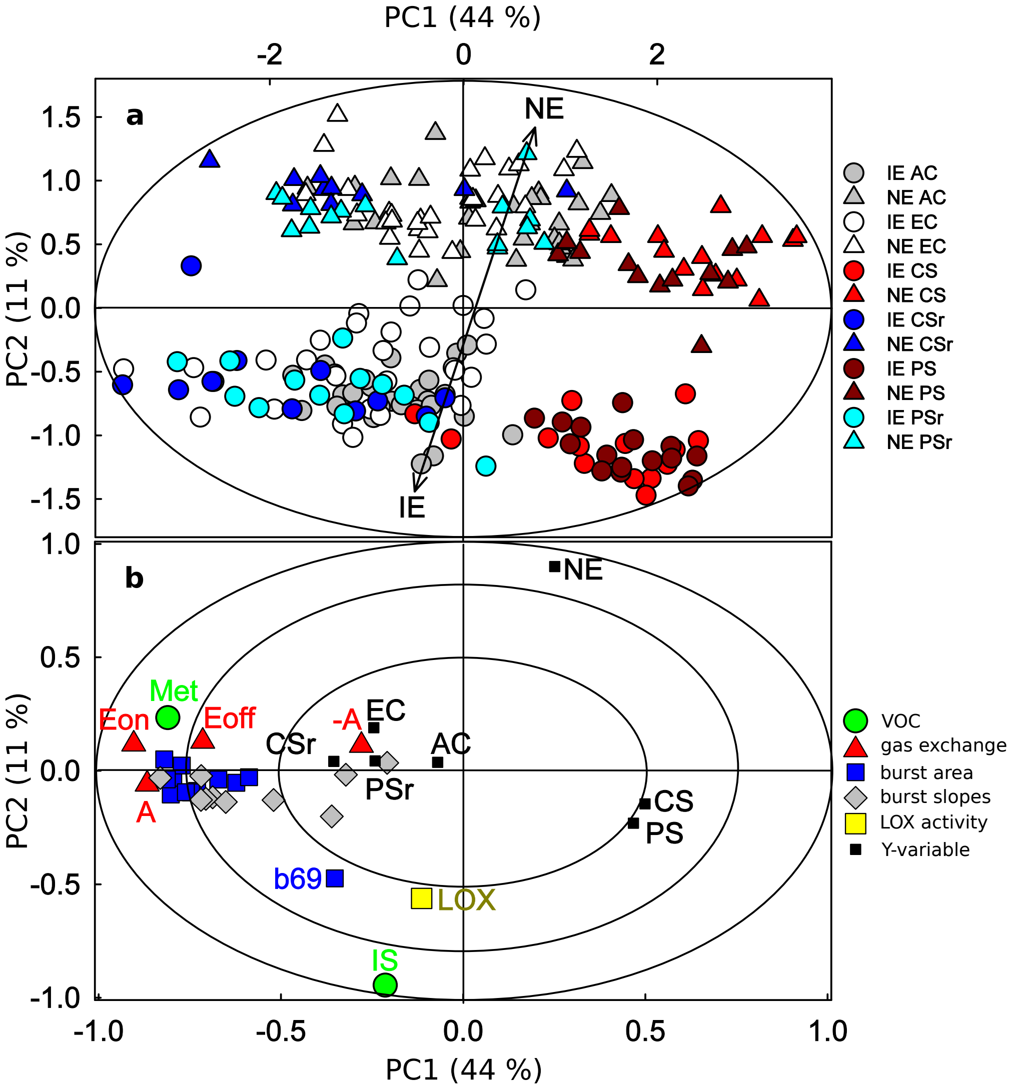

Figure S2: Score (**a**) and correlation scaled loading (**b**) plots of Orthogonal Partial Least Squares (OPLS) calculated using VOC bursts (integral of the peak curves), their slopes (through their rising edge), leaf-level VOC emission rates (of methanol and isoprene) during light conditions, gas-exchange parameters and *in-vitro* activities of LOX enzymes data. (**a**) IE (WT + PcISPS:GUS/GFP) circles; NE (RA1 + RA2) triangles; each scenario is indicated by a different colour. Each point denotes an individual sample. (**b**) Y-variables are indicated by black squares; each parameter class is indicated by a different symbol. Inner, middle and outer ellipses denote the 50, 75, 100% limits, respectively. OPLS model fits: $Q^2(Y) = 94.4\%$; $R^2(X) = 80\%$, $R^2 = 36\%$, $R^2(Y) = 67\%$ (using four PC). Abbreviations used: IE: isoprene emitter, NE: non isoprene emitter, AC: ambient control, EC: elevated control, CS: chronic stress, PS: periodic stress, CSr: chronic stress recovery, PSr: periodic stress recovery, A: net assimilation, $-A$: dark respiration, Eon: transpiration under light conditions, $Eoff$: transpiration under dark conditions, Met: methanol, IS: isoprene, $b69$: burst area isoprene.
