# Supplementary material for: Effects of heat and drought stress on post‐illumination bursts of volatile organic compounds in isoprene‐emitting and non‐emitting poplar
Source: Plant Cell Environ. 2016 Jan 18;39(6):1204–15. doi: 10.1111/pce.12643 (PMC4982041; doi:10.1111/pce.12643)
Supplement: Supplementary file 3 — Supporting info item [file PCE-39-1204-s003.pdf]

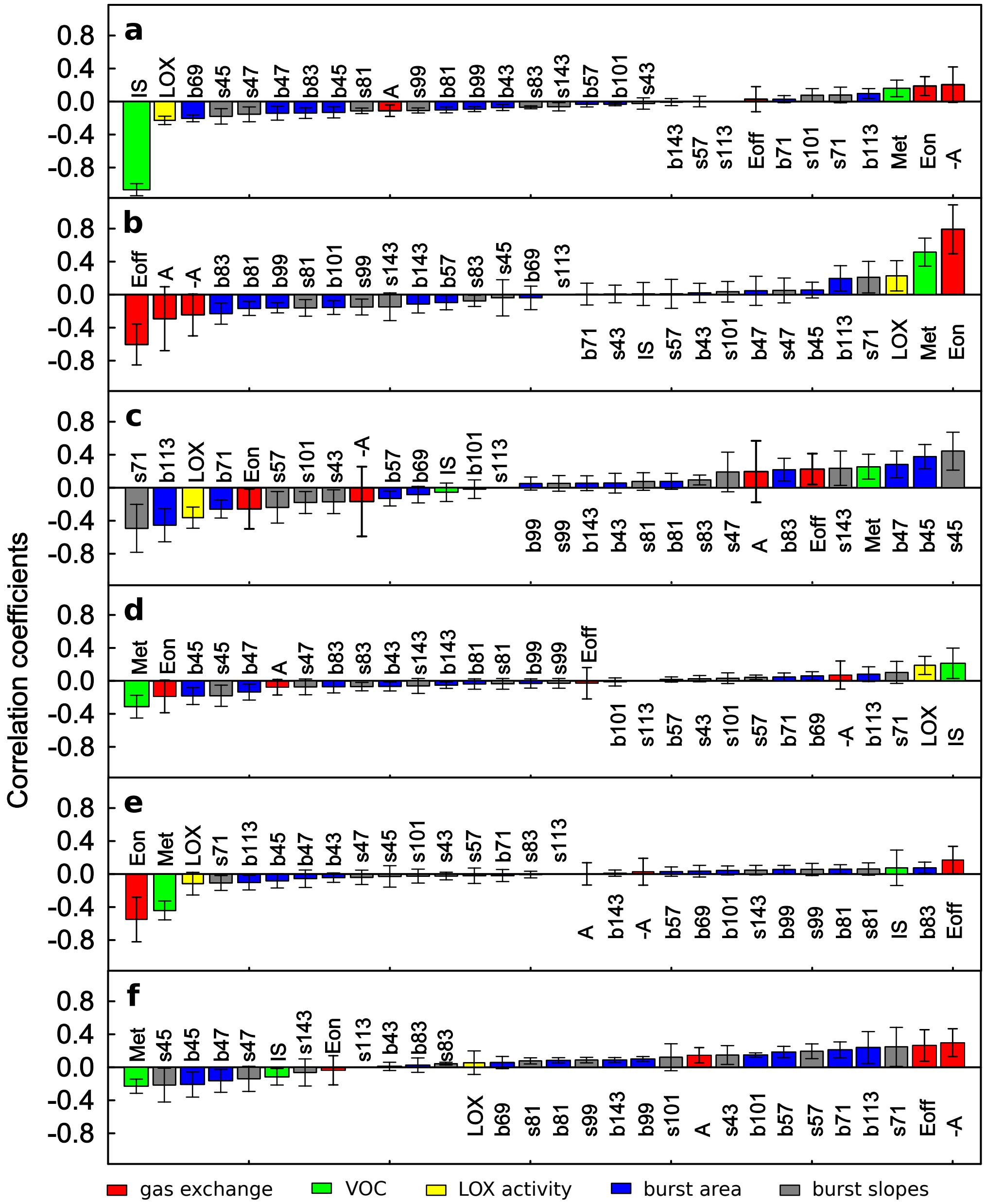

Figure S3: Correlation coefficient plots of Orthogonal Partial Least Squares (OPLS) related to respective model class information: (**a**) plant genotype, (**b**) AC, (**c**) EC, (**d**) PS, (**e**) CS, (**f**) CSr. Parameter colours follow the plot legend in Fig. S2. P-values (CV-ANOVA): (**a**) $< 1e^{-14}$, (**b**) $4.9e^{-9}$, (**c**) $2.0e^{-7}$, (**d**) $2.7e^{-13}$, (**e**) $2.7e^{-14}$, (**f**) $3.9e^{-10}$. Correlation coefficients of the PSr class were not included because the corresponding OPLS model had no significance (P = 0.36). Abbreviations used: A: net assimilation, $-A$: dark respiration, Eon: transpiration under light conditions, $Eoff$: transpiration under dark conditions, Met: methanol, IS: isoprene, s: burst slopes, b: burst area. Numbers correspond to the nominal mass of the detected ions, e.g. 45: acetaldehyde; 47: ethanol.
