# Supplementary material for: Effects of heat and drought stress on post‐illumination bursts of volatile organic compounds in isoprene‐emitting and non‐emitting poplar
Source: Plant Cell Environ. 2016 Jan 18;39(6):1204–15. doi: 10.1111/pce.12643 (PMC4982041; doi:10.1111/pce.12643)
Supplement: Supplementary file 4 — Supporting info item [file PCE-39-1204-s004.pdf]

Table S1: P-values obtained from two-way ANOVAs and Tukey post-hoc tests for the area under the post illumination bursts detected at different mass-to-charge ratios (m/z). Significant differences are marked in bold when $P < 0.05$.

Genotype	Scenario	m/z43	m/z45	m/z47	m/z57	m/z69	m/z71
IE vs. NE	AC	0.083	**<0.001**	**0.001**	0.554	**0.05**	0.406
IE vs. NE	EC	0.632	**0.01**	**0.009**	0.993	**0.032**	0.973
IE vs. NE	PS	1	0.993	1	0.948	**0.022**	1
IE vs. NE	CS	0.736	0.718	0.721	0.738	0.079	0.834
IE vs. NE	PSr	0.221	0.198	0.063	**0.003**	**0.011**	0.357
IE vs. NE	CSr	0.078	0.418	0.262	**0.013**	0.063	0.066
IE + NE	AC vs. EC	1	0.962	0.606	1	1	1
IE + NE	AC vs. PS	0.189	**<0.001**	**<0.001**	0.978	1	0.903
IE + NE	AC vs. CS	0.289	**<0.001**	**<0.001**	0.995	1	0.948
IE + NE	AC vs. PSr	0.934	0.058	0.857	**0.002**	0.997	0.535
IE + NE	AC vs. CSr	0.141	**<0.001**	**0.017**	**0.003**	1	**0.002**
IE + NE	EC vs. PS	0.118	**<0.001**	**<0.001**	0.943	1	0.916
IE + NE	EC vs. CS	0.19	**<0.001**	**<0.001**	0.979	1	0.956
IE + NE	EC vs. PSr	0.981	**0.007**	0.09	**0.003**	1	0.511
IE + NE	EC vs. CSr	0.222	**<0.001**	**<0.001**	**0.004**	1	**0.002**
IE + NE	PS vs. CS	1	1	1	1	0.998	1
IE + NE	PS vs. PSr	**0.023**	**0.017**	**0.001**	**<0.001**	1	0.091
IE + NE	PS vs. CSr	**<0.001**	0.7	0.294	**<0.001**	0.999	**<0.001**
IE + NE	CS vs.PSr	**0.042**	**0.032**	**0.002**	**<0.001**	0.988	0.125
IE + NE	CS vs. CSr	**<0.001**	0.837	0.432	**<0.001**	1	**<0.001**
IE + NE	PSr vs. CSr	0.607	0.362	0.218	1	0.994	0.146
IE	AC vs. EC	0.997	0.998	0.977	1	1	0.997
IE	AC vs. PS	0.129	**<0.001**	**<0.001**	0.97	0.999	0.856
IE	AC vs. CS	0.247	**<0.001**	**<0.001**	0.996	1	0.933
IE	AC vs. PSr	0.999	**0.007**	0.577	**0.002**	0.985	0.807
IE	AC vs. CSr	0.474	**<0.001**	**0.01**	**0.005**	1	**0.014**
IE	EC vs. PS	0.313	**<0.001**	**<0.001**	0.99	1	0.982
IE	EC vs. CS	0.506	**<0.001**	**<0.001**	0.999	0.998	0.997
IE	EC vs. PSr	0.949	**0.021**	0.193	**<0.001**	0.997	0.529
IE	EC vs. CSr	0.225	**<0.001**	**0.001**	**0.003**	1	**0.004**
IE	PS vs. CS	0.999	0.999	0.999	1	0.991	1
IE	PS vs. PSr	0.054	**0.041**	**0.003**	**<0.001**	1	0.181
IE	PS vs. CSr	**0.001**	0.71	0.321	**<0.001**	0.997	**<0.001**
IE	CS vs.PSr	0.115	0.093	**0.008**	**<0.001**	0.946	0.265
IE	CS vs. CSr	**0.003**	0.889	0.529	**0.001**	1	**0.001**
IE	PSr vs. CSr	0.72	0.565	0.352	0.999	0.97	0.237
NE	AC vs. EC	0.962	0.615	0.649	0.998	1	0.998
NE	AC vs. PS	0.967	0.278	0.279	1	1	1
NE	AC vs. CS	0.967	0.278	0.279	1	1	1
NE	AC vs. PSr	0.935	0.999	1	0.512	1	0.851
NE	AC vs. CSr	0.493	0.635	0.821	0.415	1	0.163
NE	EC vs. PS	0.597	**0.008**	**0.009**	0.984	1	0.978
NE	EC vs. CS	0.597	**0.008**	**0.009**	0.984	1	0.978
NE	EC vs. PSr	1	0.382	0.659	0.772	1	0.974
NE	EC vs. CSr	0.928	**0.037**	0.089	0.678	1	0.335
NE	PS vs. CS	1	1	1	1	1	1
NE	PS vs. PSr	0.525	0.488	0.272	0.368	1	0.682
NE	PS vs. CSr	0.131	0.989	0.934	0.286	1	0.085
NE	CS vs.PSr	0.525	0.488	0.272	0.368	1	0.682
NE	CS vs. CSr	0.131	0.989	0.934	0.286	1	0.085
NE	PSr vs. CSr	0.957	0.849	0.814	1	1	0.783

Table S1 (continued)

Genotype	Scenario	m/z81	m/z83	m/z99	m/z101	m/z113	m/z143
IE vs. NE	AC	0.353	0.491	0.327	0.427	0.405	0.29
IE vs. NE	EC	0.899	0.858	0.922	0.952	0.992	0.838
IE vs. NE	PS	0.96	0.875	0.955	1	1	1
IE vs. NE	CS	0.702	0.407	0.678	0.659	0.847	0.666
IE vs. NE	PSr	0.315	0.267	0.115	0.098	0.768	0.342
IE vs. NE	CSr	0.067	0.512	**0.023**	**0.028**	0.32	0.476
IE + NE	AC vs. EC	1	0.863	1	0.958	0.994	0.459
IE + NE	AC vs. PS	0.833	0.717	0.812	0.83	0.912	0.24
IE + NE	AC vs. CS	0.926	0.955	0.918	0.946	0.951	0.394
IE + NE	AC vs. PSr	0.527	0.14	0.17	0.071	0.851	0.271
IE + NE	AC vs. CSr	**0.002**	**<0.001**	**<0.001**	**<0.001**	0.072	**<0.001**
IE + NE	EC vs. PS	0.772	0.137	0.72	0.336	0.997	**0.003**
IE + NE	EC vs. CS	0.885	0.394	0.854	0.519	0.999	**0.007**
IE + NE	EC vs. PSr	0.602	0.723	0.231	0.355	0.537	0.999
IE + NE	EC vs. CSr	**0.002**	**<0.001**	**<0.001**	**<0.001**	**0.019**	**<0.001**
IE + NE	PS vs. CS	1	0.996	1	1	1	1
IE + NE	PS vs. PSr	0.061	**0.004**	**0.009**	**0.003**	0.275	**0.001**
IE + NE	PS vs. CSr	**<0.001**	**<0.001**	**<0.001**	**<0.001**	**0.006**	**<0.001**
IE + NE	CS vs.PSr	0.103	**0.025**	**0.018**	**0.008**	0.343	**0.003**
IE + NE	CS vs. CSr	**<0.001**	**<0.001**	**<0.001**	**<0.001**	**0.008**	**<0.001**
IE + NE	PSr vs. CSr	0.129	**0.002**	0.205	**<0.001**	0.547	**<0.001**
IE	AC vs. EC	0.998	0.994	0.757	1	0.966	0.986
IE	AC vs. PS	0.786	0.799	**0.003**	0.806	0.863	0.296
IE	AC vs. CS	0.928	0.996	0.919	0.964	0.933	0.545
IE	AC vs. PSr	0.806	0.356	**<0.001**	0.161	0.994	0.674
IE	AC vs. CSr	**0.014**	**<0.001**	0.998	**<0.001**	0.31	**<0.001**
IE	EC vs. PS	0.957	0.47	0.939	0.679	0.999	0.083
IE	EC vs. CS	0.996	0.919	0.342	0.903	1	0.202
IE	EC vs. PSr	0.537	0.686	0.342	0.244	0.756	0.959
IE	EC vs. CSr	**0.004**	**0.002**	0.993	**<0.001**	0.064	**0.004**
IE	PS vs. CS	0.999	0.982	**0.048**	0.998	1	0.998
IE	PS vs. PSr	0.136	**0.026**	**0.02**	**0.008**	0.55	**0.011**
IE	PS vs. CSr	**<0.001**	**<0.001**	0.162	**<0.001**	**0.029**	**<0.001**
IE	CS vs.PSr	0.256	0.203	**<0.001**	**0.026**	0.673	**0.033**
IE	CS vs. CSr	**0.001**	**<0.001**	**0.001**	**<0.001**	**0.046**	**<0.001**
IE	PSr vs. CSr	0.237	0.085	0.999	**0.014**	0.635	**0.038**
NE	AC vs. EC	0.989	0.893	0.985	0.926	1	0.398
NE	AC vs. PS	0.999	0.976	0.998	0.997	1	0.896
NE	AC vs. CS	0.999	0.976	0.998	0.997	1	0.896
NE	AC vs. PSr	0.844	0.616	0.718	0.623	0.88	0.605
NE	AC vs. CSr	0.133	**<0.001**	0.103	**0.001**	0.399	**<0.001**
NE	EC vs. PS	0.907	0.483	0.888	0.716	1	0.052
NE	EC vs. CS	0.907	0.483	0.888	0.716	1	0.052
NE	EC vs. PSr	0.993	0.995	0.974	0.99	0.893	0.999
NE	EC vs. CSr	0.392	**0.008**	0.351	**0.014**	0.418	**0.047**
NE	PS vs. CS	1	1	1	1	1	1
NE	PS vs. PSr	0.615	0.216	0.462	0.351	0.734	0.11
NE	PS vs. CSr	0.055	**<0.001**	**0.041**	**<0.001**	0.251	**<0.001**
NE	CS vs.PSr	0.615	0.216	0.462	0.351	0.734	0.11
NE	CS vs. CSr	0.055	**<0.001**	**0.041**	**<0.001**	0.251	**<0.001**
NE	PSr vs. CSr	0.735	**0.029**	0.799	0.062	0.958	**0.021**
