# Supplementary material for: Effects of heat and drought stress on post‐illumination bursts of volatile organic compounds in isoprene‐emitting and non‐emitting poplar
Source: Plant Cell Environ. 2016 Jan 18;39(6):1204–15. doi: 10.1111/pce.12643 (PMC4982041; doi:10.1111/pce.12643)
Supplement: Supplementary file 5 — Supporting info item [file PCE-39-1204-s005.pdf]

**Table S2:** P-values obtained from two-way ANOVAs and Tukey post-hoc tests for the LOX activity in different plant genotypes grown under the various climate scenarios. Significant differences are marked in bold when $P < 0.05$.

| **Genotype** | **Scenario** | **P** |
|---|---|---|
| IE vs. NE | AC | 0.729 |
| IE vs. NE | EC | 0.856 |
| IE vs. NE | PS | 0.372 |
| IE vs. NE | CS | 0.801 |
| IE vs. NE | PSr | 0.678 |
| IE vs. NE | CSr | 0.154 |
| IE + NE | AC vs. EC | 0.936 |
| IE + NE | AC vs. PS | 0.172 |
| IE + NE | AC vs. CS | **0.001** |
| IE + NE | AC vs. PSr | 0.999 |
| IE + NE | AC vs. CSr | 0.775 |
| IE + NE | EC vs. PS | 0.665 |
| IE + NE | EC vs. CS | **0.013** |
| IE + NE | EC vs. PSr | 0.799 |
| IE + NE | EC vs. CSr | 0.243 |
| IE + NE | PS vs. CS | 0.33 |
| IE + NE | PS vs. PSr | 0.086 |
| IE + NE | PS vs. CSr | **0.008** |
| IE + NE | CS vs.PSr | <**0.001** |
| IE + NE | CS vs. CSr | <**0.001** |
| IE + NE | PSr vs. CSr | 0.923 |
| IE | AC vs. EC | 0.975 |
| IE | AC vs. PS | 0.701 |
| IE | AC vs. CS | **0.03** |
| IE | AC vs. PSr | 1 |
| IE | AC vs. CSr | 0.676 |
| IE | EC vs. PS | 0.981 |
| IE | EC vs. CS | 0.156 |
| IE | EC vs. PSr | 0.91 |
| IE | EC vs. CSr | 0.252 |
| IE | PS vs. CS | 0.49 |
| IE | PS vs. PSr | 0.53 |
| IE | PS vs. CSr | 0.06 |
| IE | CS vs.PSr | **0.015** |
| IE | CS vs. CSr | <**0.001** |
| IE | PSr vs. CSr | 0.829 |
| NE | AC vs. EC | 0.992 |
| NE | AC vs. PS | 0.364 |
| NE | AC vs. CS | **0.038** |
| NE | AC vs. PSr | 1 |
| NE | AC vs. CSr | 0.999 |
| NE | EC vs. PS | 0.714 |
| NE | EC vs. CS | 0.136 |
| NE | EC vs. PSr | 0.969 |
| NE | EC vs. CSr | 0.93 |
| NE | PS vs. CS | 0.865 |
| NE | PS vs. PSr | 0.262 |
| NE | PS vs. CSr | 0.193 |
| NE | CS vs.PSr | **0.023** |
| NE | CS vs. CSr | **0.015** |
| NE | PSr vs. CSr | 1 |
